# Selective modes affect gene feature and function differentiation of tetraploid *Brassica* species in their evolution and domestication

**DOI:** 10.3389/fpls.2023.1142147

**Published:** 2023-04-04

**Authors:** Dayong Wei, Nan Li, Nan Zhang, Feng Liu, Jie Wu, Sa Zhao, Jinjuan Shen, Zhimin Wang, Lisha Peng, Yonghong Fan, Jiaqin Mei, Qinglin Tang

**Affiliations:** ^1^ College of Horticulture and Landscape Architecture, Southwest University, Chongqing, China; ^2^ Chongqing Yudongnan Academy of Agricultural Sciences, Mustard Tuber Research Center, Chongqing, China; ^3^ College of Agronomy and Biotechnology, Southwest University, Chongqing, China

**Keywords:** *Brassica* tetraploid, evolution, domestication, functional differentiation, positively selected genes

## Abstract

The genus *Brassica* contains a diverse group of important vegetables and oilseed crops. Genome sequencing has been completed for the six species (*B. rapa*, *B. oleracea*, *B. nigra*, *B. carinata*, *B. napus*, and *B. juncea*) in U’s triangle model. The purpose of the study is to investigate whether positively and negatively selected genes (PSGs and NSGs) affect gene feature and function differentiation of *Brassica* tetraploids in their evolution and domestication. A total of 9,701 PSGs were found in the A, B and C subgenomes of the three tetraploids, of which, a higher number of PSGs were identified in the C subgenome as comparing to the A and B subgenomes. The PSGs of the three tetraploids had more tandem duplicated genes, higher single copy, lower multi-copy, shorter exon length and fewer exon number than the NSGs, suggesting that the selective modes affected the gene feature of *Brassica* tetraploids. The PSGs of all the three tetraploids enriched in a few common KEGG pathways relating to environmental adaption (such as Phenylpropanoid biosynthesis, Riboflavin metabolism, Isoflavonoid biosynthesis, Plant-pathogen interaction and Tropane, piperidine and pyridine alkaloid biosynthesis) and reproduction (Homologous recombination). Whereas, the NSGs of the three tetraploids significantly enriched in dozens of biologic processes and pathways without clear relationships with evolution. Moreover, the PSGs of *B. carinata* were found specifically enriched in lipid biosynthesis and metabolism which possibly contributed to the domestication of *B. carinata* as an oil crop. Our data suggest that selective modes affected the gene feature of *Brassica* tetraploids, and PSGs contributed in not only the evolution but also the domestication of *Brassica* tetraploids.

## 1 Introduction

The genus *Brassica* includes several economically important species such as *B. rapa* (AA), *B. nigra* (BB), *B. oleracea* (CC), *B. napus* (AACC), *B. juncea* (AABB), and *B. carinata* (BBCC) which are widely grown around the world as fodder, vegetable, edible oil, biofuel, and condiment crops. Of the six *Brassica* species, the ancestors of three diploids (*B. rapa*, *B. nigra*, and *B. oleracea*) underwent hybridization events, giving rise to three allotetraploid species (*B. napus*, *B. juncea*, and *B. carinata*), forming the Triangle of U (Nagahara, 1935).

Many studies have illuminated the origin and evolution of A, B, and C genomes in *Brassica*. It was deduced that, before the divergence of A, B and C genomes, a common ancestor of *Brassica* was derived from *Arabidopsis thaliana* at 20-40 Mya (Beilstein et al., 2010) and experienced an extra whole-genome triplication (WGT) event at approximately 15.9–22.5 Mya (Beilstein et al., 2010; Liu et al., 2014). Consequently, the common ancestor diverged into the B genome (*B. nigra*) at about 11.5 Mya (Paritosh et al., 2021) and then the A (*B. rapa*) and C genomes (*B. oleracea*) at 4-6.8 Mya (Liu et al., 2014; Paritosh et al., 2021). Concerning to the tetraploid *Brassica* species, the A subgenome of *B. napus* might evolve from European turnip (*B. rapa*), and the C subgenome might evolve from the common ancestor of kohlrabi, cauliflower, broccoli, and Chinese kale (Lu et al., 2019), or from the *B. oleracea* cabbage-type lineage at ~2.63 Mya (Parkin et al., 2014; Yim et al., 2022). Comparison of the A subgenome in *B. juncea* with the A subgenome in *B. napus* indicated that the *B. juncea* A subgenome might derive from the ancestor of Asian *B. rapa* ssp. *tricolaris* and differ from the A subgenome in *B. napus* (Yang et al., 2016). In a recent study (Yim et al., 2022), the common ancestor of *B. juncea* and *B. carinata* diverged from the extant B genome of *B. nigra* at 6.61 Mya, and the B subgenome of *B. carinata* diverged from the *B. juncea* B subgenome at 5.44 Mya. The C subgenome of *B. carinata* is supposed to derive from the *B. oleracea* kale-type lineage at 2.88 Mya (Yim et al., 2022) thus appeared to have arisen from distinct *B. oleracea* lineage as compared with the *B. napus* C subgenome. These studies provided useful information on the origin and divergence of the A, B and C genomes of *Brassica* species, however, a more comprehensive and systematic understanding on the divergence of *Brassica* species is meaningful and available now since the release of reference genomes of all the six *Brassica* species.

Among the Brassicaceae species, *A. thaliana* was the first plant species with sequenced genome (Arabidopsis Genome, 2000) and thus is widely used as a model plant in genetic and genomic evolution studies. The six *Brassica* species in the triangle of U have also been sequenced. Among these, *B. rapa* accession Chiifu-401-42, a heading Chinese cabbage, was the first sequenced species (Wang et al., 2011), and the updated assembled version (v3.5) is now available (Zhang et al., 2022). A high-quality genome of *B. nigra* accession Ni100 v2 was recently released (Perumal et al., 2020). The genomes of two *B. oleracea* varieties, *B. oleracea* var. *capitata* line 02–12 and *B. oleracea* kale-like type TO1000 v2.1, have been assembled (Liu et al., 2014; Parkin et al., 2014). The genome of four *B. juncea*, i.e., var. *tumida* (T84−66 v1.0), T84−66 v2.0, var. *varuna* and a cultivar type (SY, yellow-seeded *B. juncea*) have also been released (Yang et al., 2016; Kang et al., 2021; Paritosh et al., 2021; Yang et al., 2021). For *B. napus*, the genome of a European winter oilseed cultivar ‘Darmor-*bzh*’ was assembled in 2014 (Chalhoub et al., 2014), and a high-quality genome assembly of version 10 was updated in 2020 (Rousseau-Gueutin et al., 2020). The high-quality genome of *B. carinata* was also published in 2021 and 2022 (Song et al., 2021; Yim et al., 2022). Owing to their agronomic importance and the availability in gaining their reference genomes, the six *Brassica* species composed an ideal model system for studying evolutionary changes in tetraploid species.

The release of reference genomes of all the six *Brassica* species not only provides the facility to investigate the divergence of *Brassica* species in depth, but also allows us to understand the evolution of *Brassica* species. In a study in *B. rapa* and *B. oleracea* (Guo et al., 2017), selective modes based on orthologous gene pairs (OGPs) between the two species, classified into positively selected genes (PSGs) and negatively selected genes (NSGs), were concerned as a determinant factor of evolutionary rates, gene compactness and expression patterns. In the present study, the divergence time (DT) of the *Brassica* genus was comprehensively and systematically estimated, and the PSGs and NSGs in *Brassica* tetraploids as comparing to corresponding diploid were investigated to understand the evolution of *Brassica* tetraploids.

## 2 Materials and methods

### 2.1 Data source

The genome sequences for *A. thaliana* (Col_v10.1), *B. rapa* (Chiifu_v3.5), *B. napus* (Darmor-bzh_v10) and *B. juncea* (T84-66_v2.0) were obtained from *Brassica* genomics database (http://brassicadb.cn). The genome data of *B. oleracea* (TO1000_v2.1) and *B. nigra* (Ni100_v2) were downloaded from EnsemblPlants (https://plants.ensembl.org/Brassica_oleracea/Info/Index) and CGI (http://cruciferseq.ca/), respectively. The genome assembly and annotation for *B. carinata* (PGL_v1) is available from Comparative Genomics platform (CoGe) under id 63922.

### 2.2 Identification of orthologs

To distinguish diverse subgenomes in *Brassica* species, we redesignated the subgenomes as follows: *B. rapa* genome as BraA, *B. nigra* genome as BniB, *B. oleracea* genome as BolC, *B. juncea* A and B subgenomes as BjuA and BjuB, respectively, *B. napus* A and C subgenomes as BnaA and BnaC, respectively, *B. carinata* B and C subgenomes as BcaB and BcaC, respectively. The interspecific all OGPs were determined using OrthoFinder2 (Emms and Kelly, 2018; Jing et al., 2023) platform (BLASTP all-*vs*-all, 10e-3, orthofinder -f./dataset -t 16 -a 8 -M msa -S blast -A mafft) in the combinations among different subgenome of *Brassica* species (BraA-BnaA, BraA-BjuA, BniB-BjuB, BniB-BcaB, BolC-BnaC and BolC-BcaC) and combinations between *A. thaliana* and *Brassica* species (Ath-BraA, Ath-BniB and Ath-BolC). The rates of nonsynonymous substitution (Ka) and the rates of synonymous substitution (Ks) values for each pair of homologous genes were estimated using ParaAT 2.0 (https://bigd.big.ac.cn/tools/). Genes with Ks > 1 were discarded, since such high Ks values may imply either potential sequence saturation or misalignment. The divergence time was calculated with the formula T = Ks/2λ, where λ is the neutral substitution rate of 8.22e–09 synonymous substitutions per site per year for Brassicaceae species (Beilstein et al., 2010).

### 2.3 Selective mode and functional enrichment analysis

Almost all the protein-coding genes can be classified into three categories: PSGs, NSGs, and neutral genes. To differentiate these genes, the Ka/Ks ratio was calculated for each gene, and Ka/Ks > 1, Ka/Ks = 1, and Ka/Ks < 1 were used as the indicators for positive selection, neutral evolution and negative selection (from small to large, the same number with PSGs) during gene sequence divergence, respectively. The physical location distribution of PSGs and NSGs were visualized by ‘CMplot’ R package (the widows size was 1 Mb).

Gene Ontology (GO) enrichment analysis was performed by ‘topGO v2.50.0’ package (https://bioconductor.org/packages/release/bioc/html/topGO.html) with R-4.1.2 software (significant genes≥5 and the Kolmogorov-Smirnov test ‘KS’<0.05) and TBtools v1.09876 software (GO level≥4, hits genes≥5 and P-value<0.01) (Chen et al., 2020). Kyoto Encyclopedia of Genes and Genomes (KEGG) pathway was analyzed using ‘clusterProfiler v4.2.2’ package (Wu et al., 2021) with R-4.1.2 software (gene count≥5 and P-value<0.05) and TBtools v1.09876 software (hits genes≥5 and P-value<0.05). These subgenomic OGPs of tetraploid *Brassica* species were used for background genes, and their function annotation were predicted by eggNOG-mapper v2 online service (http://eggnog-mapper.embl.de/) (Cantalapiedra et al., 2021).

### 2.4 Statistical analysis

The one-tailed test through a random sampling way was used to investigate whether PSGs affect the domestication of *Brassica* tetraploids. In details, for an interested tetraploid (for example *B. carinata*), the PSGs enriched in a given biologic process related to domestication was considered as gene set 1 (90 PSGs specifically enriched in lipid-related pathway in this study), and reported genes associating with the same biologic process was considered as gene set 2 (2,507 reported lipid-related genes). Firstly, the number of common genes overlapped between gene set 1 and 2 was found out and denoted by *N_com_* (21 intersecting genes). Then, a same number genes as gene set 1 (or gene set 2) were randomly chosen from all annotated genes of this tetraploid (90 or 2,507 genes randomly choose from 133,235 annotated gene), and then looked for the intersection with gene set 2 (or gene set 1). A total of 100,000 runs was carried out for the two algorithms, resulted in 50,000 intersections for each. The threshold of the right tail in the 99% confidence interval was calculated for each algorithm (50,000 intersections) as the mean gene number of corresponding 50,000 intersections plus 2.33*S.D (mean+2.33*S.D., 4.68). (Rosner, 2015). Finally, *N_com_* was compared with the threshold of the right tails calculated for the two algorithms (4.68). If *N_com_* falls outside of the 99% confidence intervals, the PSGs in gene set 1 will be considered to play a positive role in the domestication of the given biologic process. Other descriptive statistics were performed using Excel 2021. Analysis of variance (ANOVA) and Duncan’s multiple range test (DMRT) were carried out *via* IBM SPSS Statistics software v25 (https://www.ibm.com/products/spss-statistics).

## 3 Results

### 3.1 The distribution and characteristics of Ka, Ks, and Ka/Ks

A total of 561,662 OGPs was detected from the six combinations among *Brassica* species (BraA-BnaA, BraA-BjuA, BniB-BjuB, BniB-BcaB, BolC-BnaC and BolC-BcaC) and three combinations between *A. thaliana* and *Brassica* species (Ath-BraA, Ath-BniB and Ath-BolC). After discarding 112,332 genes with Ks>1 and Ka=0, the values of Ka, Ks and Ka/Ks were calculated for the remaining 449,330 gene pairs (**Table 1**). The Ka/Ks exhibited approximately normal distributions (single peak curves) in all the nine combinations (**Figure S1**). A significant positive correlation was found between Ka and Ks in each of the nine combinations (*r*=0.70~0.71, *P*<0.01). The Ka/Ks ratio was moderately correlated with Ka (*r*=0.12~0.26, *P*<0.01), while it showed a slight negative correlation with Ks (*r*=-0.14~-0.27, *P*<0.01), indicating that Ka is the determinant factor for Ka/Ks.

**Table 1:** Comparisons of Ka, Ks, and Ka/Ks.

Category^1^	Annotated genes	Pair of OGPs	Ka^2^	Ks	Ka/Ks
Ath-BraA	27416-47,250	30,968	0.101 ± 0.074 A	0.532 ± 0.173 A	0.202 ± 0.125 C
Ath-BniB	27,416-59,852	34,817	0.112 ± 0.083 A	0.531 ± 0.183 A	0.203 ± 0.133 C
Ath-BolC	27,416-59,221	33,436	0.111 ± 0.081 A	0.543 ± 0.183 A	0.201 ± 0.131 C
BraA-BnaA	47,250-47,193	55,877	0.073 ± 0.085 B	0.299 ± 0.266 B	0.293 ± 0.299 B
BraA-BjuA	47,250-47,895	55,871	0.072 ± 0.083 B	0.301 ± 0.264 B	0.292 ± 0.317 B
BniB-BjuB	59,852-46,785	60,752	0.070 ± 0.083 B	0.291 ± 0.259 B	0.282 ± 0.284 B
BniB-BcaB	59,852-77,290	71,423	0.067 ± 0.081 B	0.268 ± 0.238 B	0.287 ± 0.294 B
BolC-BnaC	59,221-59,692	57,120	0.079 ± 0.090 B	0.304 ± 0.270 B	0.350 ± 0.366 A
BolC-BcaC	59,221-65,365	49,066	0.073 ± 0.084 B	0.282 ± 0.248 B	0.342 ± 0.345 A

^1^Ath, Arabidopsis genome; BraA, *B. rapa* genome; BniB, *B. nigra* genome; BolC, *B. oleracea* genome; BjuA, A subgenomes of *B. juncea*; BnaA, A subgenomes of *B. napus*; BjuB, B subgenomes of *B. juncea*; BcaB, B subgenomes of *B. carinata*; BnaC, C subgenomes of *B. napus*; BcaC, C subgenomes of *B. carinata*.

^2^Mean ± SD, and different capital letters represent significant differences at P<0.01

Both the Ka and Ks of each *A. thaliana*–*Brassica* combination were significantly higher than those of the combinations of different *Brassica* species (DMRT, *P*<0.01). The mean Ka/Ks ratio of the *A. thaliana*–*Brassica* combinations was significantly lower than that of the within-*Brassica* combinations (**Table 1**). These results indicate that the *A. thaliana* genes possibly underwent a higher evolutionary rate and experienced more selective pressure than the *Brassica* genes.

### 3.2 Gene feature analyses of PSGs and NSGs

A total of 9,701 PSGs were found in all combinations, with an average of 1,617 pairs in each combination. A higher number of PSGs were identified in the combination of C subgenome (average 2,028 pairs) than in A and B subgenomes (average 1373 and 1451 pairs) (**Table 2**). This indicates that more genes of *B. oleracea* (CC) were positively selected during the evolution of *B. napus* (AACC) and *B. carinata* (BBCC). Unevenly distribution of PSGs and NSGs were found in all chromosomes, and the NSGs are far from the centromere (**Figure S2**).

**Table 2:** The PSGs and NSGs in subgenome combinations among *Brassica* species.

Category^1^	OGPs^2^	Ka/Ks^3^	
		Rank	Mean ± SD
PSGs			
BraA-BnaA	1,372	1.000-11.13	1.510 ± 0.731
BraA-BjuA	1,373	1.000-18.27	1.525 ± 0.972
BniB-BjuB	1,310	1.000-11.21	1.507 ± 0.691
BniB-BcaB	1,591	1.000-13.15	1.490 ± 0.832
BolC-BnaC	2,296	1.000-12.39	1.557 ± 0.852
BolC-BcaC	1,759	1.000-12.08	1.520 ± 0.801
NSGs			
BraA-BnaA	1,372	0.002-0.025	0.016 ± 0.006
BraA-BjuA	1,373	0.002-0.024	0.015 ± 0.005
BniB-BjuB	1,310	0.002-0.021	0.014 ± 0.005
BniB-BcaB	1,591	0.002-0.022	0.015 ± 0.005
BolC-BnaC	2,296	0.002-0.047	0.029 ± 0.011
BolC-BcaC	1,759	0.003-0.042	0.025 ± 0.010

^1^: PSGs, Positively selected genes; NSGs, Negatively selected genes; BraA, *B. rapa* genome; BniB, *B. nigra* genome; BolC, *B. oleracea* genome; BjuA, A subgenomes of *B. juncea*; BnaA, A subgenomes of *B. napus*; BjuB, B subgenomes of *B. juncea*; BcaB, B subgenomes of *B. carinata*; BnaC, C subgenomes of *B. napus*; BcaC, C subgenomes of *B. carinata*.

^2^: OGPs, Orthologous gene pairs.

^3^: Ka, The rates of nonsynonymous substitution; Ks, The rates of synonymous substitution.

To understand whether and how selective modes affect gene features, the tandem duplication, the copy number, the exon length and number of these PSGs and NSGs were characterized. It was found that PSGs had a significantly more tandem duplicated genes (average 5.47% *vs*. 1.68%), higher single copy (average 99.43% *vs*. 65.01%), lower multi-copy (average 0.28% *vs*. 17.50%), shorter exon length (average 1051 bp *vs*. 1798 bp) and fewer exon number (average 5.1 *vs*. 7.0) than the NSGs (**Table 3**), revealing obvious varied gene features between PSGs and NSGs. This finding indicated that selective modes may serve as an alternative indicator for gene compactness.

**Table 3:** Gene feature analysis between PSGs and NSGs.

Variables^1^	PSGs			NSGs			P-value (t-test)
	*B. napus*	*B. juncea*	*B. carinata*	*B. napus*	*B. juncea*	*B. carinata*	
Single copies	99.62%	99.22%	99.46%	61.56%	67.68%	65.79%	4.53E-05
Double copies	0.38%	0.67%	0.54%	26.06%	21.92%	25.37%	4.88E-05
> Triple copies	0%	0.11%	0%	12.38%	10.4%	8.84%	5.11E-04
Exon length (bp)	954	1040	1158	1303	1424	2667	3.89E-17
Exon number	4.8	4.9	5.7	6.6	7.3	7.0	4.39E-18
TDGs	4.91%	6.34%	5.16%	1.47%	1.53%	2.03%	6.49E-03

^1^ TDGs: Tandem duplicated genes.

### 3.3 The divergence time of *Brassica* species

**Figure 1:**
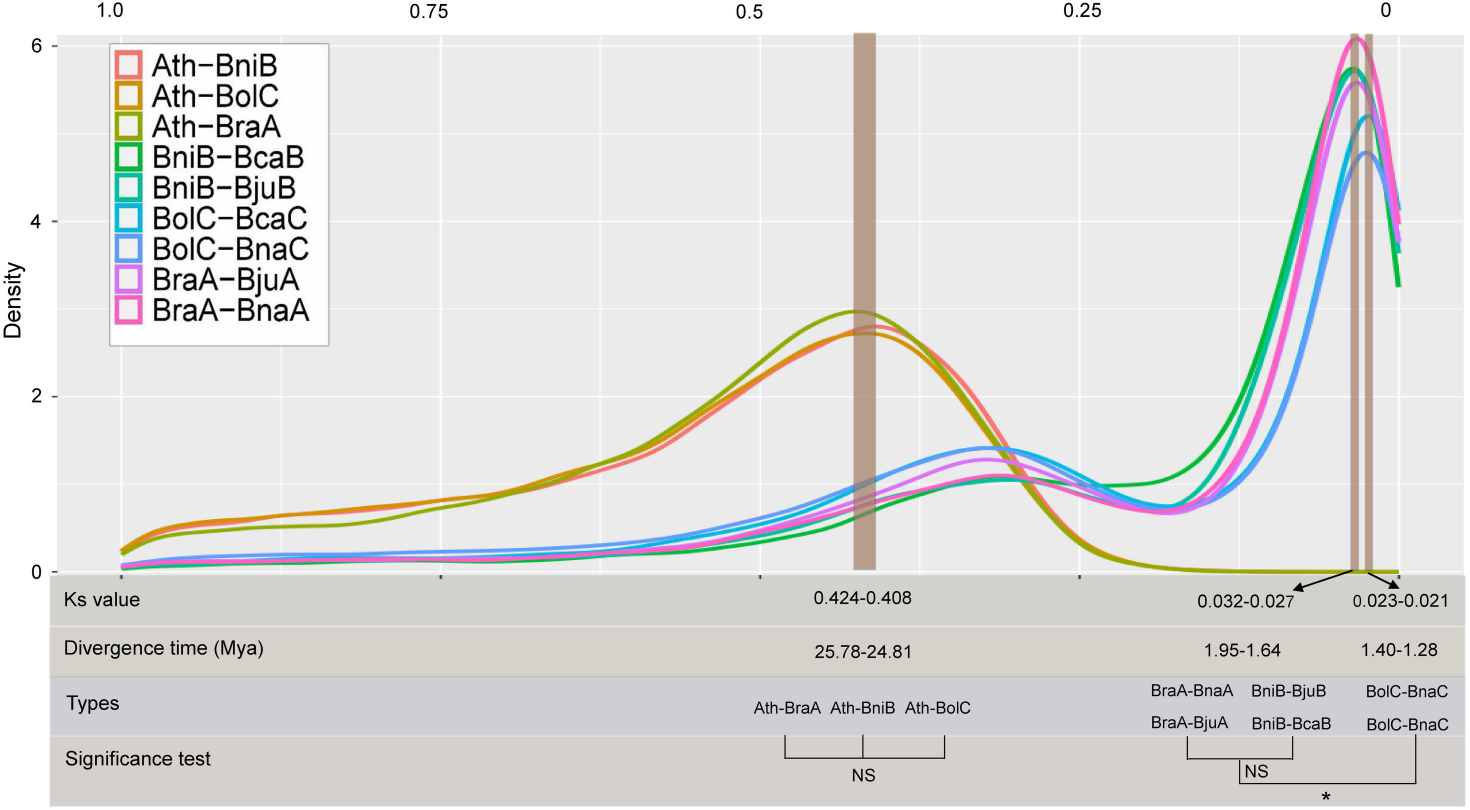
Divergence time estimation based on Ks distributions among different combinations. NS: no significance; *: significance level at 0.05. Ath, *Arabidopsis* genome; BraA, *B rapa* genome; BniB, *B nigra* genome; BolC, *B oleracea* genome; BjuA, A subgenome of *B juncea*; BnaA, A subgenome of *B napus*; BjuB, B subgenome of *B juncea*; BcaB, B subgenome of *B carinata*; BnaC, C subgenome of *B napus*; BcaC, C subgenome of *B carinata*.

To comprehensively and systematically estimate the divergence time of the *Brassica* species, we investigated the distribution of the Ks in nine combinations. The Ks peak between *A. thaliana* and diploid *Brassica* species (*B. rapa*, *B. oleracea* and *B. nigra*) was 0.408–0.424, indicating that the *A. thaliana* and diploid *Brassica* species separated at ~ 24.81–25.78 Mya. In similar, by estimating the peak Ks values in the A subgenomes (BraA-BnaA, BraA-BjuA), B subgenomes (BniB-BjuB, BniB-BcaB) and C subgenomes (BolC-BnaC, BolC-BcaC) combinations, the divergence time of subgenomes was speculated at ~1.27–1.96 Mya. Of which, significant differences were found between A and C subgenomes (average 1.77 *vs* 1.29 Mya, *P*=0.042), between B and C subgenomes (average 1.91 *vs* 1.29 Mya, *P*=0.0068), but no significant difference was detected between A and B subgenomes (average 1.77 *vs* 1.91 Mya, *P*=0.31) (**Figure 1**).

### 3.4 Functional differentiation between PSGs and NSGs

**Figure 2:**
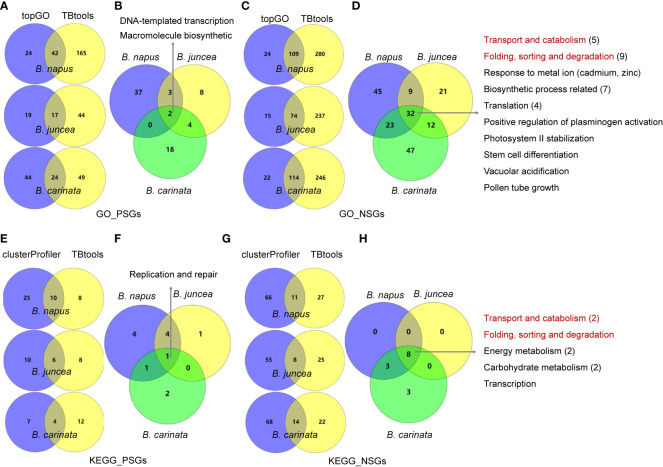
GO enrichment and KEGG pathway analysis for PSGs and NSGs in the evolution of tetraploid *B napus*, *B juncea* and *B carinata*. **(A, C)**: GO enrichment analysis for PSGs and NSGs in three tetraploid *Brassica* using topGO and TBtools software, three intersecting terms were showed in middle; **(B, D)**: two and 32 common terms were found in three tetraploid *Brassica*; **(E, G)**: KEGG pathway analysis for PSGs and NSGs in three tetraploid *Brassica* by clusterProfiler and TBtools software, three intersecting terms were showed in middle; **(F, H)**: one and eight common pathways were found in three tetraploid *Brassica*. The red font represent same terms between GO and KEGG.

**Figure 3:**
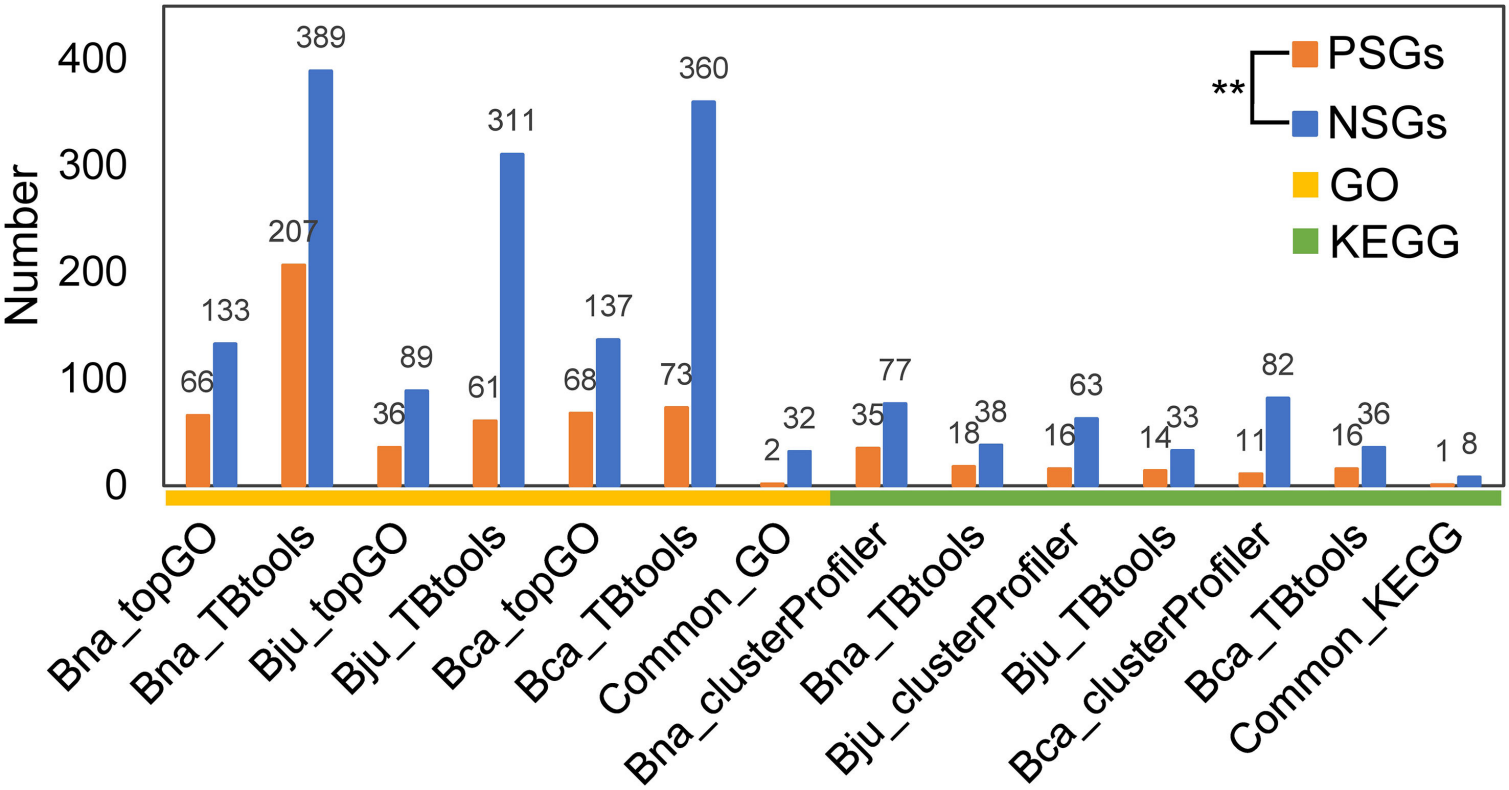
The number of GO terms and KEGG pathways for PSGs and NSGs. **significance level at 0.01. Bna, *B.napus*; Bju, *B juncea*; Bca, *B carinata*.

To understand the functional differentiation of genes involved in the evolution from diploids to tetraploids, we performed functional enrichment analyses on the PSGs and NSGs in A, B and C subgenomes. By GO enrichment analyses through two methods, the PSGs of *B. napus* (BraA-BnaA and BolC-BnaC), *B. juncea* (BraA-BjuA and BniB-BjuB) and *B. carinata* (BniB-BcaB and BolC-BcaC) were significantly enriched in 42, 17 and 24 biological processes, respectively (**Figure 2A**). Among these terms, only two (GO:0006355, regulation of DNA-templated transcription and GO:2000112, regulation of cellular macromolecule biosynthetic process) were commonly enriched in all the three tetraploids (**Figure 2B**; **Table S1-S3**). In contrast, these NSGs were significantly enriched in 32 common terms among the three tetraploids, including five terms relating to transport and catabolism, nine associating with folding, sorting and degradation, two relating to response to metal ion, four relating to translation, seven associating with biosynthetic process and five unclassified terms (**Figure 2C, D**; **Table S1-S3**). In similar, the KEGG pathway analysis found that the PSGs were enriched in only one common pathway (B09124: Replication and repair) in the three tetraploids (**Figure 2E, F**; **Table S4-S6**), whereas, five common pathways (B09141: Transport and catabolism; B09123: Folding, sorting and degradation; B09102: Energy metabolism; B09101: Carbohydrate metabolism and B09121: Transcription) were significantly enriched by the NSGs of these tetraploids (**Figure 2G, H**; **Table S4-S6**). For each tetraploid *Brassica*, the PSGs enriched in less GO terms and KEGG pathways than the NSGs (average 85 *vs*. 237 GO terms, and 18 *vs*. 55 KEGG pathways) (**Figure 3**). The data may indicate that functional differentiation has occurred between PSGs and NSGs in the evolution of *B. napus*, *B. juncea* and *B. carinata*.

### 3.5 The contribution of PSGs in the evolution and domestication of tetraploids

In total, ten, five and four KEGG pathways were enriched by the PSGs of *B. napus*, *B. juncea* and *B. carinata*, respectively (**Figure 2F**), with one common pathway “Homologous recombination” (ko03440) which is a secondary class of “Replication and repair” (B09124). This result indicates that genes associating with reproduction was positively selected in all the three tetraploids. On the other hand, four, one and two pathways were specifically enriched in *B. napus*, *B. juncea* and *B. carinata* respectively, however, most of these pathways were related to environmental adaptation. In detail, the PSGs of *B. napus* were specifically enriched in Starch and sucrose metabolism (ko00500), Cyanoamino acid metabolism (ko00460), Phenylpropanoid biosynthesis (ko00940) and Riboflavin metabolism (ko00740), of which the last two are related resistance to biotic or abiotic stresses. In similar, the PSGs of *B. juncea* were exclusively enriched in Isoflavonoid biosynthesis (ko00943), and Plant-pathogen interaction (ko04626) and Tropane, piperidine and pyridine alkaloid biosynthesis (ko00960) were specifically enriched in *B. carinata.* Our data suggest that PSGs play important roles in the evolution of tetraploids.

**Figure 4:**
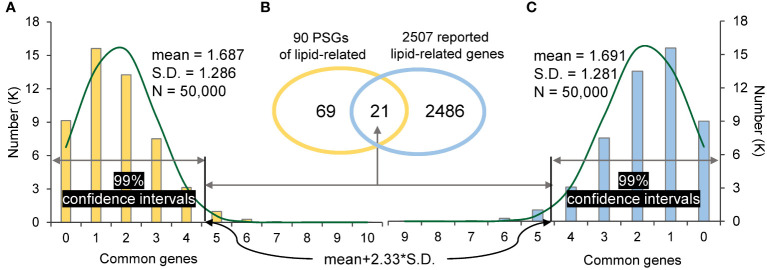
The random sampling distribution and significance test. **(A)**: the distribution of common genes between 90 random sampling genes and 2507 reported lipid-related genes. **(B)**: The common genes between 90 PSGs of lipid-related and 2507 reported lipid-related genes. **(C)**: the distribution of common genes between 2507 random sampling genes and 90 PSGs of lipid-related. The threshold of the right tail is ‘mean+2.33*S.D.’ in the 99% confidence interval.

In addition, PSGs were also found to contribute to domestication. For example, *B. carinata*, an ancient crop from the Ethiopian highlands (Yim et al., 2022), is mainly domesticated as a crop with desirable seed fatty acid for biofuel production. In this study, 90 PSGs (gene set 1) of *B. carinata* were found specifically enriched in lipid biosynthesis (GO:0008610) and lipid metabolism (GO:0006629) (**Table S7**). In order to know whether PSGs affect lipid-related genes in *B. carinata* domestication, 2507 reported lipid-related genes of *B. carinata* (gene set 2) (Yim et al., 2022) were subjected to the one-tailed test as described in “Materials and Methods”. A total of 21 common genes (*N_com_*) was found between gene set 1 and gene set 2, which fell outside of the two 99% confidence intervals (the right tails were 4.684 and 4.678 for the two algorithms, respectively) (**Figure 4**). This suggests that lipid-related genes were positively selected in *B. carinata* domestication history.

## 4 Discussion

### 4.1 The evolution and domestication route of the *Brassica* genus

**Figure 5:**
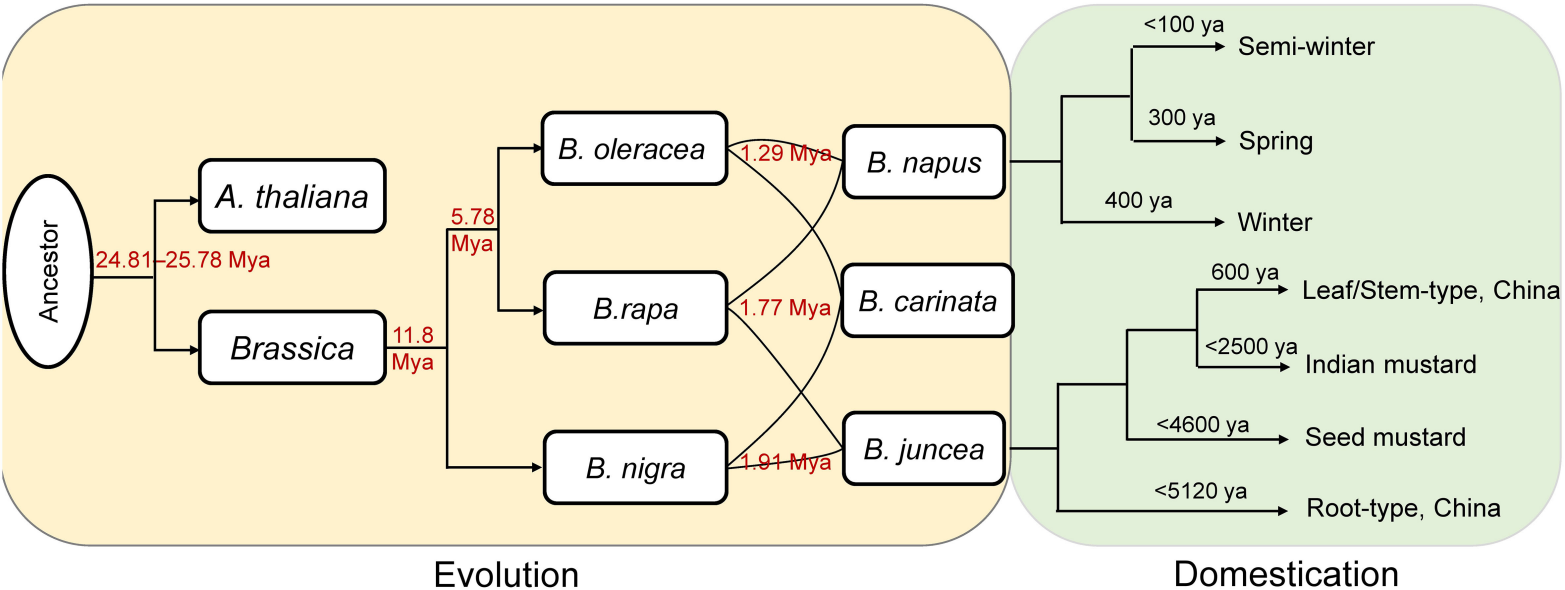
The evolution and domestication history. The red numbers were calculated in this study. ya, years ago.

Combined with previous reports, a framework was provided here to illustrate the evolution and domestication of the *Brassica* genus (**Figure 5**). Based on the comprehensive and systematic estimation of the divergence time of the *Brassica* species according to Ks values, the *Brassica* species might be diverged from *A. thaliana* at about 24.81–25.78 Mya. This is in accordance with a previous study which found the *Brassica* lineage diverged from *A. thaliana* at about 29.5 Mya (Song et al., 2021). The divergence time was earlier than the hexaploidization event (23 Mya) and the known time of the WGT event (15.9–22.5 Mya) specific to *Brassica* (Beilstein et al., 2010; Liu et al., 2014). Based on the single-copy genes, we deduced that the divergence time of subgenomes at 1.27~1.96 Mya, which was significantly larger than that previously reports (0.043–0.076 Mya) using colinear genes (Song et al., 2021), however, consistent result was found that the formation time of B subgenomes was earlier than that of C subgenomes.

Concerning to the diploid *Brassica* species, the divergence time among *B. rapa*, *B. oleracea*, and *B. nigra* estimated in the present study was generally consistent with other studies. For example, *B. rapa* and *B. oleracea* were proposed to have diverged from *B. nigra* at ~11.5 Mya (Parkin et al., 2014; Paritosh et al., 2021), and here we deduced that *B. rapa* and *B. oleracea* split from *B. nigra* at ~11.8 Mya (**Figure 5**). Likewise, the divergence time between *B. rapa* and *B. oleracea* was reported to be 4–6.8 Mya (Parkin et al., 2014; Paritosh et al., 2021), and we found the two species might diverge from each other at ~5.78 Mya (**Figure 5**).

Concerning to the tetraploid *Brassica* species, *B. napus* was found originate from the spontaneous hybridization between *B. rapa* and *B. oleracea* (Lu et al., 2019). The winter type (the original type) and the spring type of *B. napus* were formed at ~400 and ~300 ya, respectively, while the semi-winter type was domesticated more recently (<100 ya) (Qian et al., 2006; Bonnema, 2012; Wei et al., 2017). Recent research found that *B. juncea* most likely have a single origin from West Asia at about 8,000–14,000 ya (Kang et al., 2021). The earliest cultivation of *B. juncea* occurred at 2,500–5,120 ya in China as a vegetable crop (root type), followed by domestication of the seed mustard in West and Central Asia at 1,800–4,600 ya. The seed mustard spread from southern Afghanistan into the Indian subcontinent where it was domesticated into Indian mustard about 2,500 ya, and then spread further east into southwestern China at ~600 ya, formed a the broad-leaf/stem type *B. juncea* (Kang et al., 2021).

### 4.2 Selection bias of PSGs in subgenomes of *Brassica*

In this study, the number of PSGs was significantly greater in the C subgenome (average 2028) than in the A and B subgenomes (average 1412), and had no correlation with the subgenome size of *Brassica* (r=0.442, *P*=0.386). This result indicated that more genes of *B. oleracea* (CC) were positively selected during the evolution of *B. napus* (AACC) and *B. carinata* (BBCC). It is in consistence with previous studies that the C subgenome has undergone stronger selection in the evolution of *B. napus* and *B. carinata* (Wei et al., 2017; An et al., 2019; Yim et al., 2022). This could partially express the narrow genetic diversity of C subgenomes in *B. napus* and *B. carinata*, and therefore, introduce of novel genetic diversity into the C subgenomes is of great importance in broaden the genetic diversity of the two tetraploids. It is well known that *B. oleracea* has rich genetic variation (at least six cultivated and more than ten wild taxa) (Snogerup et al., 1990; Gladis and Hammer, 2001) and carries many favorable genes such as stress tolerance or disease resistance genes (Ellis et al., 1999; Mei et al., 2010; Wu et al., 2013; Wei et al., 2014). Although the two tetraploids and *B. oleracea* are cross-incompatibility, embryo rescue technique could solve this problem in a certain degree and it is possible to introduce genetic components from *B. oleracea* into *B. napus* or *B. carinata via* a hexaploid bridging strategy (Mei et al., 2015).

### 4.3 Roles for PSGs in the evolution and domestication of tetraploid *Brassica*

Positive selection and negative selection are considered as two major modes of natural selection (Xu et al., 2015). However, their influences on the evolutionary force of allotetraploids are still poorly understood. In the present study, we found obvious functional differentiation between PSGs and NSGs of tetraploid *B. napus*, *B. juncea* and *B. carinata*. It was found that PSGs of all the three tetraploids were significantly enriched in pathways associating with reproduction (Homologous recombination) and environmental adaptation. The role of homologous recombination in the evolution and domestication of *Brassica* allotetraploids has been well documented (Chalhoub et al., 2014), as well as in several other species (Bertioli et al., 2019; Edger et al., 2019; Zhang et al., 2020). The ability of environmental adaptation is a well-known factor in natural selection. Furthermore, we found lipid-related genes were positively selected in *B. carinata*. As we know, *B. carinata* is a crop with desirable seed fatty acid for biofuel production. Comparatively, this trait (seed fatty acid) seems more related to artificial selection rather than natural selection. Overall, our investigation suggests that positive selection is an important driving force in not only the evolution but also the domestication of *Brassica* tetraploids. Our study provides valuable insights into the selective mode and the evolution and domestication of *Brassica* tetraploid species.

## Data availability statement

The original contributions presented in the study are included in the article/**Supplementary Material**. Further inquiries can be directed to the corresponding authors.

## Author contributions

DW, JM and QT conceived and designed the experiments. NL, NZ and FL collected and analyzed the data. SZ, NZ, JW, LP, JS and ZW performed software analysis. YF and QT reviewed the manuscript. DW and NL wrote the manuscript; all authors reviewed and approved the final manuscript. All authors contributed to the article and approved the submitted version.
