# Supplementary material for: Selective modes affect gene feature and function differentiation of tetraploid Brassica species in their evolution and domestication
Source: Front Plant Sci. 2023 Apr 4;14:1142147. doi: 10.3389/fpls.2023.1142147 (PMC10110867; doi:10.3389/fpls.2023.1142147)
Supplement: Supplementary file 2 [file DataSheet_1.docx]

**Figure S1** The frequency distribution of Ka/Ks in different combinations

Ath, *Arabidopsis* genome; BraA, *B. rapa* genome; BniB, *B. nigra* genome; BolC, *B. oleracea* genome; BjuA, A subgenomes of *B. juncea* ; BnaA, A subgenomes of *B. napus*; BjuB, B subgenomes of *B. juncea*; BcaB, B subgenomes of *B. carinata*; BnaC, C subgenomes of *B. napus*; BcaC, C subgenomes of *B. carinata.*

**Figure S2** The physical location distribution for PSGs and NSGs in all chromosomes.

The grey oval represents the centromeres from Yim et al., 2022; Kang et al., 2021; Rousseau-Gueutin et al., 2020. Bna, *B.napus*; Bju, *B. juncea*; Bca, *B. carinata*; PSGs, Positively selected genes; NSGs, Negatively selected genes. The widows size was 1 Mb.
